# Modulation of Ricin Intoxication by the Autophagy Inhibitor EACC

**DOI:** 10.3390/toxins14050360

**Published:** 2022-05-22

**Authors:** Kirsten Sandvig, Simona Kavaliauskiene, Anne Grethe Myrann, Tore Geir Iversen, Tore Skotland

**Affiliations:** 1 Department of Molecular Cell Biology, Institute for Cancer Research, Oslo University Hospital, The Norwegian Radium Hospital, 0379 Oslo, Norway; simona.kavaliauskiene@medisin.uio.no (S.K.); anne.grethe.myrann@rr-research.no (A.G.M.); t.g.iversen@ous-research.no (T.G.I.); toresko@uio.no (T.S.); 2 Department of Biosciences, University of Oslo, 0315 Oslo, Norway; 3 Institute for Experimental Medical Research, University of Oslo and Oslo University Hospital Ullevål, 0450 Oslo, Norway

**Keywords:** ricin, abrin, volkensin, viscumin, modeccin, plant toxins, autophagy, Golgi apparatus, endoplasmic reticulum, retrograde transport

## Abstract

The compound EACC (ethyl (2-(5-nitrothiophene-2-carboxamido) thiophene-3-carbonyl) carbamate) was recently reported to inhibit fusion of autophagosomes with lysosomes in a reversible manner by inhibiting recruitment of syntaxin 17 to autophagosomes. We report here that this compound also provides a strong protection against the protein toxin ricin as well as against other plant toxins such as abrin and modeccin. The protection did not seem to be caused by inhibition of endocytosis and retrograde transport, but rather by inhibited release of the enzymatically active A-moiety to the cytosol. The TANK-binding kinase 1 (TBK1) has been reported to phosphorylate syntaxin 17 and be required for initiation of autophagy. The inhibitor of TBK1, MRT68601, induced in itself a strong sensitization to ricin, apparently by increasing transport to the Golgi apparatus. Importantly, MRT68601 increased Golgi transport of ricin even in the presence of EACC, but EACC was still able to inhibit intoxication, supporting the idea that EACC protects at a late step along the retrograde pathway. These results also indicate that phosphorylation of syntaxin 17 is not required for the protection observed.

## 1. Introduction

It is becoming increasingly clear that a number of cellular processes such as endocytosis, degradation, and anterograde as well as retrograde transport in the Golgi apparatus and the ER are subject to complex regulation by signaling [1,2]. Thus, when attempting to study regulation of one specific process by knockdown of certain proteins or by manipulation of SNARE complexes or their phosphorylation status, it is challenging to obtain a complete overview of the cellular responses and the changes induced.

Protein toxins such as the plant toxin ricin and the bacterial toxin Shiga toxin have proven valuable to investigate changes in several intracellular transport steps [3]. These toxins consist of two moieties, one that binds to the cell surface and another moiety that inhibits protein synthesis by enzymatically removing one adenine from the 28S RNA of the 60S ribosomal subunit after entry into the cytosol [3,4,5,6]. In the case of ricin, the toxin binds to both glycoproteins and glycolipids with terminal galactose, whereas Shiga toxin binds to the neutral glycosphingolipid Gb3. Both toxins are endocytosed by different endocytic mechanisms, and they are then transported to the Golgi apparatus and the ER before the enzymatically active subunit is released and translocated across the ER membrane. Importantly, the toxins seem to differ when it comes to regulation of their entry into cells and the retrograde transport [3]. Not only do they differ when it comes to the kinetics of retrograde transport, but also the proteins and lipid species required for transport are different, with differences that can be exploited to study the cellular processes involved and their regulation.

It was recently reported that the compound EACC is a specific inhibitor of fusion between autophagosomes and lysosomes [7] and that this compound prevented recruitment of the SNAREs syntaxin 17 (Stx17) and SNAP29 onto the autophagosomal membrane. It also inhibited the interaction of Stx17 with the HOPS subunit Vps33A and the SNARE VAMP8. Furthermore, the TANK-binding kinase 1 (TBK1) controls autophagy initiation by binding to and phosphorylating Stx17 [8]. Since Stx17 has also been reported to be present in the ER [9], the ERGIC [10], and the Golgi apparatus [8,10], and to be involved in ER dynamics [9], we wanted to investigate whether the compound EACC and an inhibitor of TBK1, MRT68601 [11], affects retrograde toxin transport and intoxication. EACC might be an important tool also to investigate ER function, although its exact target is not yet known. 

As demonstrated in the current article, EACC can provide a very good protection against the plant toxin ricin, a protection which seems to be related to the inhibited release of ricin A-chain after toxin entry into the ER. Unexpectedly, the TBK1 inhibitor strongly increased ricin transport to the Golgi apparatus, sensitized the cells to ricin, and thereby counteracted the protective effect of EACC. However, EACC was still able to protect against ricin in the presence of MRT68601. EACC protected different cell types also against other plant toxins, such as abrin, modeccin, viscumin, and volkensin, demonstrating that it may have a general effect on translocation of plant toxins from the ER. In contrast, there was only a very small protection on the effect of Shiga toxin, supporting the view that the cellular mechanisms for entry are not identical.

## 2. Results

### 2.1. EACC Inhibits the Toxic Action of Ricin 

**Figure 1:**
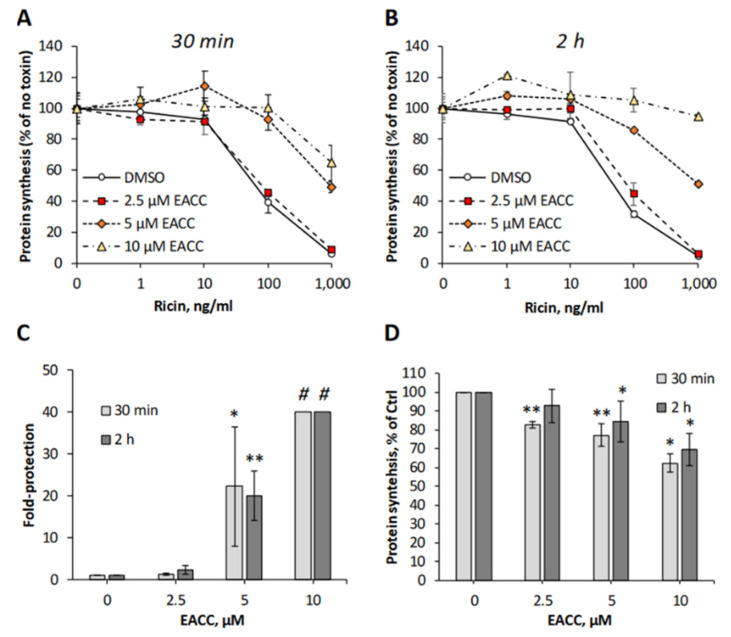
EACC protected HEp-2 cells against ricin. HEp-2 cells were treated with EACC for 30 min (**A**) or 2 h (**B**) and subsequently incubated with increasing concentrations of ricin for 3 h, before protein synthesis was measured. Representative experiments are shown (**A**,**B**). (**C**) Fold protection was calculated as an increase in IC50 in EACC-treated samples compared to control (DMSO-treated cells). *#:* At 10 µM dose, cells were fully protected against ricin (for up to 1000 ng/mL) and protection could not be calculated. (**D**) Protein synthesis in the cells treated with EACC for 3.5 h (30 min + 3 h when the toxin was added to other wells) or 5 h (2 h + 3 h when toxin was added to other wells). *n* ≥ 3; * *p* ≤ 0.05, ** *p* ≤ 0.005.

To investigate whether addition of EACC to cells was able to affect any of the steps used by ricin on its way to the cytosol, we mimicked the conditions of autophagy induction by removing the serum and incubating HEp-2 cells with different concentrations of EACC for increasing time, before ricin was added and the protein synthesis was measured 3 h later. Even when ricin was added as soon as after a 30 min preincubation with EACC, the cells were well-protected against ricin (Figure 1A,C), and after a 2 h preincubation, the protection was even better (Figure 1B,C). At a 10 µM concentration of EACC, the protection was so good that we could not measure 50% inhibitory concentration of ricin in the concentration range used (Figure 1C). Importantly, EACC was tolerated well by the cells and had only a minor effect on total protein synthesis (Figure 1D). The protective effect of EACC was abolished in the presence of serum, and as reported for its inhibitory effect on fusion between autophagosomes and lysosomes [7], the protection against ricin was also reversed upon its removal from the medium (not shown). Similarly, as shown for HEp-2 cells, EACC also protected PC3 cells and HeLa cells against ricin (Figure S1).

### 2.2. Ricin Is Transported All the Way to the ER in the Presence of EACC

**Figure 2:**
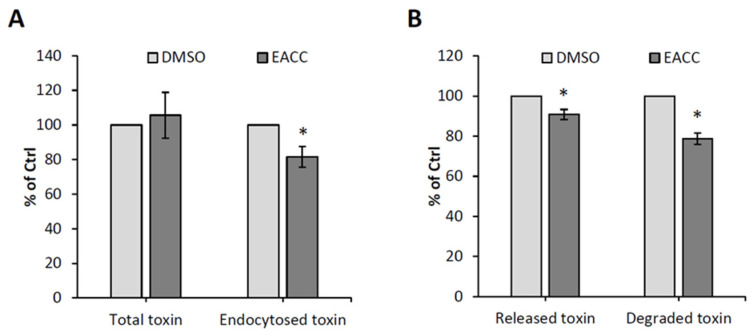
The effects of EACC on ricin binding, uptake, and recycling. HEp-2 cells were treated with 0.1% DMSO or 5 µM EACC for 2 h followed by incubation with ^125^I-ricin for 20 min. The ricin endocytosis (**A**) and recycling and degradation (**B**) were measured; *n* = 3, as described in Materials and Methods. Endocytosed toxin was measured after 20 min (**A**), and released and degraded toxin was measured after an additional 2 h chase; * *p* ≤ 0.05.

To investigate which step in ricin entry that was inhibited by EACC, we first measured binding to the cell surface and endocytic uptake by using ^125^I-labeled ricin. As shown in Figure 2A, there was no effect on binding to the cell surface, but there was a slight (20%) inhibition of the endocytic uptake. Additionally, there was a small decrease in recycling back to the cell surface and in degradation of ricin (Figure 2B), which could be due to reduced ricin uptake after EACC treatment. 

**Figure 3:**
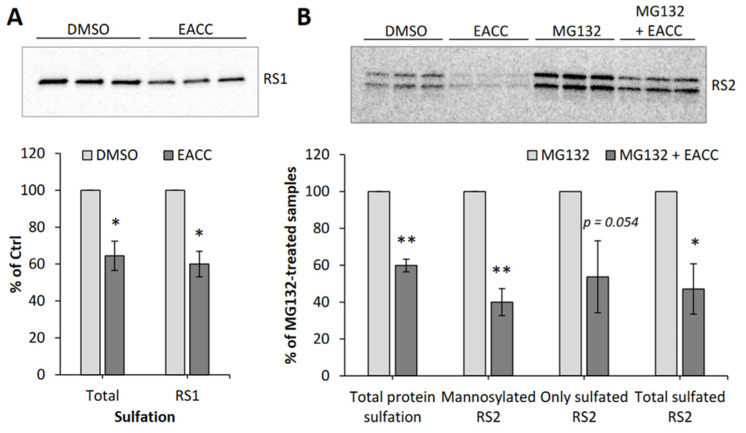
Effect of EACC on sulfation of ricin and endogenous proteins. (**A**) HeLa cells were treated with 0.1% DMSO or 5 µM EACC for 2 h and then were subjected to sulfation assay with ricin-sulf1 (RS1). The upper figure shows an autoradiogram of one representative experiment, and the lower figure shows the quantification of total protein sulfation and RS1 sulfation expressed as percent of control (DMSO); *n* = 3. (**B**) HEp-2 cells were treated with 0.1% DMSO or 5 µM EACC for 2 h and then were subjected to sulfation assay with ricin-sulf2 (RS2). To half of the wells, proteasome inhibitor MG132 (10 μM) was added together with RS2. The upper figure shows an autoradiogram of one representative experiment, and the lower figure shows the quantification of RS2 sulfation and mannosylation expressed as percent of MG132 treated cells; *n* = 3. Only sulfated RS2 is the lower band. When the sulfated RS2 was also mannosylated, it moved more slowly; that is the upper band, and the columns called total sulfated RS2 are the sum of the two bands. In all these experiments, the cells were incubated for 1 h with ^35^SO_4_^2−^ before addition of inhibitors. After addition of the ricin constructs, the incubation was continued for 3 h before removal of surface-bound toxin, lysis, immunoprecipitation, electrophoresis, Western blotting, and quantification of the radioactive bands; * *p* ≤ 0.05, ** *p* ≤ 0.005.

To investigate ricin transport to the Golgi and ER, we used two different ricin constructs, one with a sulfation site in the A-chain (RS1), and another with a sulfation site and a glycosylation site as well (RS2). We added radioactive sulfate to obtain labeling of the toxin constructs (both RS1 and RS2) in the trans-Golgi by sulfotransferases [12]. There is a shift in the molecular weight of the sulfated A-chain in RS2 when it is modified by ER enzymes. After addition of EACC, sulfation of RS1 was somewhat reduced (Figure 3A), but it was reduced to a similar extent as sulfation of endogenous cellular molecules (total protein sulfation), suggesting that RS1 transport to the Golgi may not be affected much by EACC. It seems unlikely that the very good protection against ricin provided by EACC was due to inhibition of ricin transport to the Golgi. When using the RS2 construct, the challenge was that the radioactive signal from radioactive sulfate became split into two bands (only sulfated and sulfated plus glycosylated) and then became quite weak, especially after addition of EACC (Figure 3B). We therefore added the proteasome inhibitor MG132 to some cells to prevent degradation, and as shown in Figure 3B, the signals then became much stronger. The compound MG132 had in itself no effect on ricin toxicity or on the protection afforded by EACC (Figure S2). Importantly, the data clearly showed that EACC does not prevent the formation of the high molecular weight second band, which was previously demonstrated to be the glycosylated form of the A-chain [12]. Examples of sulfation experiments with RS1 and RS2 and quantification of the bands are shown in Figure 3. The data indicate that ricin is transported all the way to the ER even in the presence of EACC. Thus, an inhibited retrograde transport does not seem to be the explanation for the strong EACC-induced protection. 

### 2.3. EACC Inhibits Release of Ricin A-Chain 

**Figure 4:**
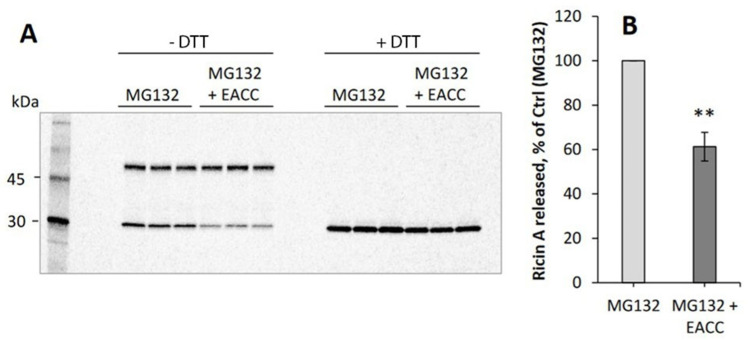
Release of ricin A-chain after EACC treatment. HEp-2 cells were incubated for 1 h with ^35^SO_4_^2−^ and then EACC was added to some of the cells (5 μM), and the incubation was continued for 2 h more before further addition of RS1 and MG132 (10 µM) to all the cells. After 3 h of additional incubation, N-ethylmaleimide (1 mM) was added to prevent further reduction of RS1, and surface-bound toxin was removed with lactose. Then the cells were lysed, and an anti-ricin A-chain antibody was used for immunoprecipation. The precipitates were then washed twice with PBS containing TritonX-100 (0.35%), resuspended in Laemmli sample buffer with and without 100 mM DTT, and boiled for 5 min before being run on SDS-PAGE. Further processing and quantification were performed as described in Materials and Methods. In (**A**) is shown one representative example of such an experiment where the samples to the left were run on a non-reducing gel (−DTT) and the samples to the right on a reducing gel (+DTT). (**B**) shows quantification of the release of A-chain from independent experiments (*n* = 3); ** *p* ≤ 0.005.

To intoxicate cells, it is not sufficient that ricin is able to reach the ER; the A-chain has to be released from the B-chain by reduction of the disulfide bond connecting the two chains, and it also has to be translocated to the cytosol. To investigate whether the release of A-chain is affected by EACC, we again used RS1, the ricin construct with a sulfation site in the A-chain. The cells were incubated with this construct in the presence of radioactive sulfate with and without EACC, and at the end of the incubation, N-ethylmaleimide was added to the cells to prevent further reduction which might otherwise happen during the subsequent lysis of the cells. Ricin A-chain and intact ricin were immunoprecipitated, and then run on an SDS-gel under non-reducing and reducing conditions. As shown in Figure 4A to the left (non-reducing gel), EACC inhibited formation of the lower band which represents the free A-chain. The figure also demonstrates that in this experiment, there was little difference in total sulfated A-chain when the gel was run under reducing conditions. Quantification of independent experiments is shown in Figure 4B. These experiments were performed in the presence of the proteasome inhibitor MG132 to prevent proteasomal degradation, otherwise the bands became extremely weak. Thus, the data indicate that EACC inhibits ER-mediated reduction of ricin. It has recently been shown that ER function and exit of proteins from the ER are strongly dependent on signaling, and both adenylyl cyclase and the leukocyte tyrosine kinase (LTK), which is an ER-resident tyrosine kinase, affect transport out of the ER [1,13,14]. However, neither the addition of the membrane permeable cAMP analogue 8-Br-cAMP nor the inhibition of LTK by addition of crizotinib (Figure S3) had any effect on the ability of EACC to protect the cells against ricin or on the sensitivity of the cells to the toxin alone. 

### 2.4. The TBK1 Inhibitor MRT68601 Increases Ricin Transport to the Golgi and Sensitizes Cells to Ricin 

**Figure 5:**
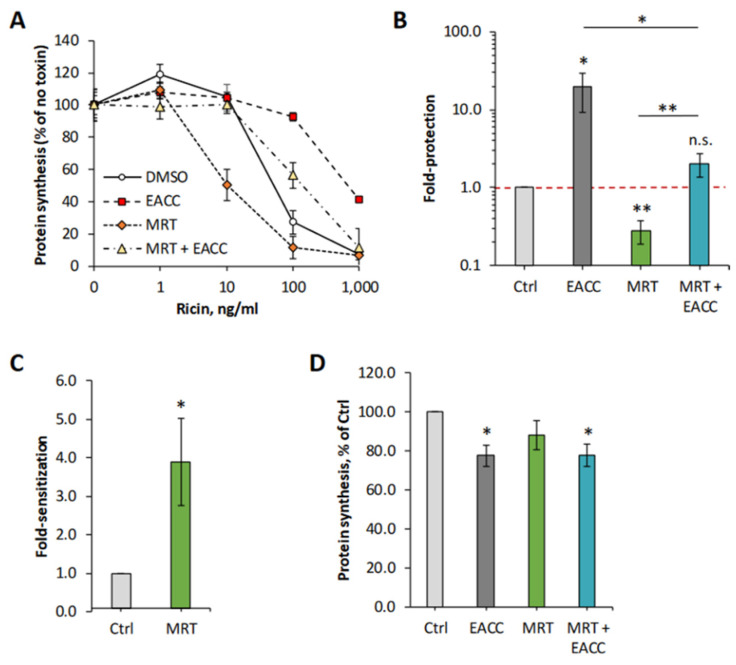
MRT68601 strongly sensitized cells to ricin, an effect counteracted by EACC. HEp-2 cells were treated with 25 µM MRT68601 or DMSO (0.1%) for 30 min prior to addition of 5 µM EACC or DMSO. Following additional 2 h incubation with the inhibitors, the cells were incubated with increasing concentrations of ricin for 3 h, before protein synthesis was measured. One representative experiment is shown in (**A**). (**B**) Fold protection was calculated as an increase in IC50 in EACC-treated samples compared to control (DMSO-treated cells). (**C**) Fold sensitization was calculated for the samples treated with MRT alone. (**D**) Protein synthesis in the cells treated with the inhibitors for 5.5 h (2.5 h + 3 h when the toxin was added to other wells). *n* = 4. Protection by EACC and sensitization by MRT68601 were significant compared to DMSO, while the combination of the two was not significantly different from the DMSO; * *p* ≤ 0.05, ** *p* ≤ 0.005.

As described in the introduction, autophagy initiation is dependent on the kinase TBK1 [8], and we therefore tested whether an inhibitor of this kinase would affect the protective effect seen with EACC on HEp-2 cells. As shown in Figure 5, MRT68601 (25 µM) surprisingly induced a strong sensitization to ricin. However, EACC was still able to induce a good protection in the presence of MRT68601. A representative experiment is shown in Figure 5A, and the graphs in Figure 5B,C show a quantification of the protection and sensitization from independent experiments. As also demonstrated (Figure 5D), none of the treatments had any major effect on protein synthesis. As expected, the ability of MRT68601 to sensitize cells to ricin was concentration dependent, as shown in Supplementary Figure S4. 

**Figure 6:**
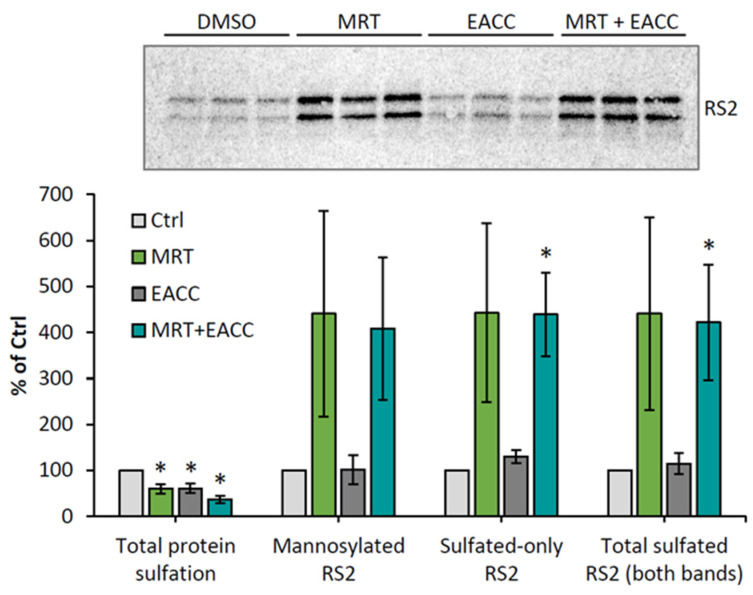
MRT68601 increased ricin transport to Golgi and ER also when combined with EACC. HEp-2 cells were first incubated for 30 min with ^35^SO_4_^2−^ and then the cells were treated with 25 µM MRT68601 or 0.1% DMSO for 30 min prior to addition of 5 µM EACC or 0.1% DMSO. After further incubation for 2 h, ricin-sulf2 (RS2) was added and the incubation was continued for 3 h more before removal of surface-bound toxin, lysis of the cells, immunoprecipitation of the toxin, and further processing as described earlier. The upper figure shows an autoradiogram of one representative experiment, and the lower figure shows the quantification of RS2 sulfation and mannosylation expressed as percent of DMSO-treated cells, *n* = 3; * *p* ≤ 0.05.

To investigate which step of ricin transport was affected by MRT68601, we again used the ricin construct RS2 with both a sulfation site and a glycosylation site, and we investigated what happens both with MRT68601 alone and in combination with EACC. As shown in Figure 6, MRT68601 strongly increased the sulfation of this construct, indicating an increased transport of the toxin to the Golgi apparatus. There was an equally strong increase also in the glycosylation of ricin as indicated by the heavier sulfated band, and the data thus suggested that the efficiency of trans-Golgi to ER transport was unchanged; the increase in transport to the Golgi seemed to be reflected by a similar increase in transport to the ER. Importantly, even in the presence of both MRT68601 and EACC, glycosylation occurred to the same extent as with MRT alone (Figure 6), indicating that even though more ricin entered the ER than in control cells, EACC was able to inhibit transfer of ricin into the cytosol. Additionally, in HeLa cells, sulfation experiments showed that MRT68601 in a concentration-dependent manner increased ricin transport to the Golgi apparatus (Figure S5). 

The increased transport of ricin to the Golgi in the presence of MRT68601 could be due to increased endosome to Golgi transport and/or to increased endocytic uptake of the toxin. However, experiments performed with ^125^I-labeled ricin demonstrated that MRT68601 slightly decreased the endocytic uptake; thus, the increase in Golgi transport seems to occur from the endosomal compartment (Figure S5). Concerning the endocytic compartments, both EACC and MRT68601 did have some effect on transferrin binding and endocytosis and seemed to affect endocytosis and/or recycling of this ligand (Figure S6). Preincubation of the cells with EACC reduced the available transferrin-binding sites on the cell surface (measured as transferrin binding at 4 °C) and also had a slight effect on the endocytic uptake after short time (5 min). After a longer incubation (20 min), the fraction of endocytosed transferrin was higher than in control cells, suggesting that recycling was inhibited. In the case of MRT68601, a preincubation with this drug followed by incubation with transferrin at a low temperature revealed an increased transferrin binding to the cell surface. In this case, a short incubation with transferrin at 37 °C (5 min) showed a reduced endocytic uptake, a finding that may explain the increase in cell surface binding of transferrin. Even in this case, there may be a small inhibitory effect on recycling as the internalized fraction increased somewhat with time. However, also after a longer incubation with transferrin, the fraction of endocytosed ligand did not increase above that of the control. Thus, there seems to be a pleiotropic effect of these compounds on the endocytic compartment, but the increase in Golgi transport seen with MRT68601 far exceeded the minor effects on endocytosis and recycling. 

### 2.5. Ability of EACC and MRT68601 to Affect Toxicity of Other Protein Toxins

**Figure 7:**
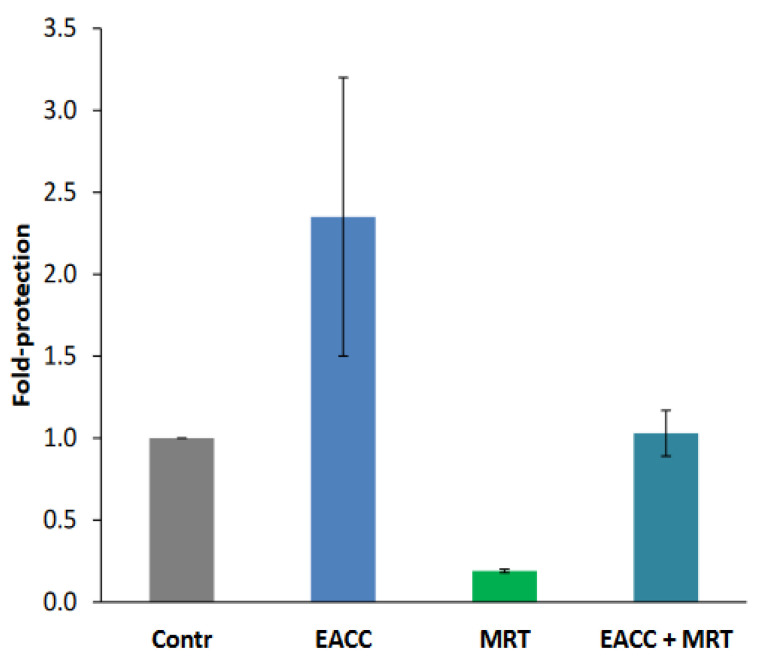
EACC protected and MRT68601 sensitized to Shiga toxin. HEp-2 cells were washed once with HEPES-buffered medium and then treated with 25 µM MRT or 0.1% DMSO for 30 min at 37 °C. Then, 5 µM EACC or DMSO (0.1%) was added and the incubation was continued for 2 h. Finally, the cells were incubated with increasing concentrations of Shiga toxin for 4 h, before protein synthesis was measured. Fold protection was calculated as fold change in IC50 in the inhibitor-treated samples compared to control (Ctrl; DMSO-treated cells). *n* = 2.

**Figure 8:**
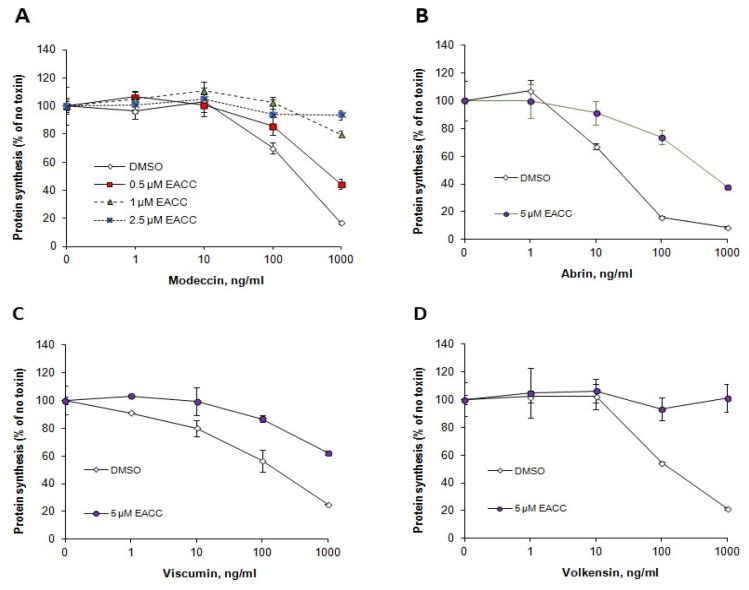
EACC protected against modeccin (**A**), abrin (**B**), viscumin (**C**), and volkensin (**D**). HEp-2 cells were treated with or without EACC for 2 h, and then incubated with the indicated concentrations of the various toxins for 3 h. The error bars show deviations between duplicates in a representative experiment. *n* = 3.

Like ricin, Shiga toxins are also transported retrogradely to the ER before release and translocation of their enzymatically active moiety to the cytosol. However, their requirements for retrograde transport differ from those of ricin, and their translocation from the ER to the cytosol may also require different ER components. We therefore tested whether EACC and MRT68601 had any effect also on the sensitivity of cells to Shiga toxin. As shown (Figure 7), EACC seemed to give a slight protection against Shiga toxin on HEp-2 cells, and this protection was counteracted by MRT68601, which in itself sensitized the cells to the toxin. EACC also protected against the plant toxins abrin, modeccin, viscumin, and volkensin (Figure 8). Thus, the protection against ricin afforded by EACC was similar for a number of other plant toxins. 

## 3. Discussion

The most striking result in the present article was the ability of the compound EACC to induce a strong protection against ricin, as well as against other plant toxins. The data indicate that for the toxin we focused on in this study, ricin, this is mainly due to an inhibition of release of ricin A-chain in the ER, possibly also coupled to a decrease in translocation from the ER to the cytosol, since the protection seems to be stronger than the inhibition of reductive release of A-chain from the B-chain. Due to the sensitivity of the assay, we could not measure release after short times of incubation, raising the question of whether there is a change in the kinetics of reduction and translocation of the A-chain that might explain the larger effect on toxicity than on A-chain release measured after 3 h. EACC had little effect on endocytic uptake of ricin, there did not seem to be any strong effect on transport to the Golgi, and the results indicated that transport between the trans-Golgi and the ER was unchanged. Clearly, the data showed that a sulfated ricin construct, which also contained an ER glycosylation site, was modified by ER enzymes. As expected, there was no further modification of a ricin construct without this glycosylation site. 

In agreement with our data showing that EACC had very little effect on ricin endocytosis and degradation in HEp-2 cells is the reported lack of effect of EACC on EGF-induced degradation of the EGF-receptor and the lack of conversion of procathepsin B to mature cathepsin B in HeLa cells [7]. Since EACC was reported to inhibit the association of autophagosomes with Stx17 [7] and this SNARE has to be phosphorylated by TBK1 to initiate autophagy [8], we tested whether inhibition of TBK1 with MRT68601 would affect the protection afforded by EACC. As shown, it did counteract the protection, but this may not be related to the effect of EACC as the compound in itself sensitized the cells to ricin. Surprisingly, this sensitizing effect seemed to be due to a strong increase in transport of ricin from endosomes to the Golgi apparatus, whereas there was no further effect on transport from trans-Golgi to the ER. Importantly, addition of EACC gave a strong protection when added together with MRT68601 (compared to after addition of MRT68601 only), supporting the idea that EACC protects at a later step than transport to the ER. Thus, this is in agreement with the view that reduction and translocation of ricin is the step that is affected by EACC.

The reason for the MRT68601-induced increased transport of ricin from endosomes to the Golgi is not obvious. However, it is now known that there are a number of common effectors in the endocytic and autophagic machinery [15], and it was found that TBK1 is acting upstream of recruitment of proteins involved in autophagy to endosomes [11]. Inhibition of recruitment of these factors was found to inhibit recycling of the EGF-receptor through the Rab11-positive recycling compartment, whereas no effect was found on the rate of uptake of the EGF-receptor or on uptake and recycling of the transferrin receptor. An effect of the TBK1-inhibitor MRT68601 on endosomal recruitment of proteins might be related to the change in transport of ricin to the Golgi apparatus. It is possible that when recycling is inhibited by inhibition of TBK-1, transport in the direction of the Golgi apparatus might be facilitated. In future studies, it would be valuable to investigate the endosomal protein complexes known to affect Golgi transport under these conditions and to study whether transport of other ligands and membrane components moving in the direction of the Golgi apparatus are also affected. 

An important question is why there is an inhibition of ricin A-chain release and translocation from the ER in the presence of EACC. Whether reduction of the disulfide bond in ricin is coupled to its translocation to the cytosol is not known, and it is possible that the protection afforded by EACC could directly affect the translocation machinery in the ER as well as the reduction of the disulfide bond. It was previously reported that inhibition of transport out of the ER by expression of a negative Sar1 mutant protected against ricin [16], but whether the release of the A-chain was also affected was not investigated at that time. It was also suggested [17] that interfering with ER kinases that regulate transport out of the ER might affect the translocation of protein toxins to the cytosol. The tyrosine kinase LTK was reported to interact with Sec12, a GEF for Sar1, and thereby serve as a regulator of ER exit site biogenesis [2,14]. However, the inhibitor of LTK, crizotinib, which was shown to retard transport out of the ER [14], did not inhibit the toxicity of ricin nor did it have any effect on the protection afforded by EACC. Similarly, addition of 8-Br-cAMP, which also affects ER signaling and transport [13], had no effect on intoxication with ricin. A compound reported to inhibit transport of syntaxin 5 out of the ER is Retro-2 [18,19]. However, the action of EACC seems to be different from that of Retro-2 as EACC does not protect against ricin in the presence of serum, and under serum starvation, the protection against ricin is very good, in contrast to what is seen with Shiga toxin, results which are opposite for Retro-2. Retro-2 was found to protect strongly against Shiga toxin by preventing movement of the Shiga toxin binding protein GPP130 from endosomes to the Golgi apparatus [18]. In the case of EACC, there did not seem to be any change in colocalization of syntaxin 5 with GM130, and there also was no change in colocalization of GPP130 with TGN46 (our unpublished results).

The protection afforded by EACC is not expected to be related to Stx17, as it was reported that depletion of Stx17 did not affect neither anterograde transport of VSVG-GFP nor retrograde transport of VSVG-KDEL-R-YFP [10]. However, there may be an effect on exit of other proteins that might be important for reduction and translocation of ricin to the cytosol. Stx17 interacts with p24 proteins, proteins known to interact with cargos leaving the ER [10]. Additionally, Stx17 is required for a normal structure of the ERGIC and the Golgi apparatus. It cannot be excluded that structural changes of the ER in themselves might change the interaction of ricin with endogenous proteins involved in its reduction and cytosolic entry [20]. Both ricin and Shiga toxin have been reported to interact with Sec61 [21,22,23,24]. However, since there was such a large protection against ricin but essentially no protection against Shiga toxin, one would not expect that Sec61 was the target of EACC. In agreement with that, immunolabeling of Sec61α (and also BIP) did not reveal any large changes (our unpublished results). Now that the cellular localization for interference with ricin entry caused by EACC seems to be localized to the ER, future studies can be concentrated on the ER proteins possibly affected. An increased understanding of EACC-induced changes can provide us with a better understanding of regulation of transport in the ER. 

## 4. Materials and Methods

### 4.1. Reagents and Antibodies

Unless otherwise stated, the chemicals were bought from Sigma-Aldrich. Ricin-sulf1 and ricin sulf-2 were made as previously described [12]. EACC was bought from Life Chemicals and MRT68601 was obtained from Tocris. Modeccin [25] and abrin [26] were purified as previously described, and Shiga toxin (Stx) was provided by Dr. J. E. Brown (USAMRIID, Fort Detrick, MD, USA) and Dr. J. Kozlov (Academy of Science of Russia, Moscow, Russia). H_2_^35^SO_4_ (S-RA-1) was purchased from Hartmann Analytic. ^125^I (NEZ033A010MC) for protein labeling, and [^3^H]leucine (NET460005MC) was from Perkin Elmer. Rabbit anti-ricin and anti-ricin A were home-made [27].

*Cell culture*. HEp-2 cells (CCL-23) and HeLa cells were from ATCC (Manassas, VA, USA) and both cell types were grown in Dulbecco’s Modified Eagle Medium (DMEM, D0819, Sigma-Aldrich, Saint Louis, MO, USA), with 10% fetal serum (FBS, F7524, Sigma-Aldrich, Irvine, United Kingdom). PC-3 cells (CRL-1435) were from ATCC (Manassas, VA, USA) and were grown in DMEM/F-12 (1:1) (31331-093, Thermo-Fisher Scientific) with 7% FBS. All cells were grown in medium supplemented with 100 U/mL penicillin and 100 μg/mL streptomycin (P4333, Sigma-Aldrich, Saint Louis, MO, USA) at 37 °C and 5% CO_2._

### 4.2. Protein Synthesis Measurements

When protein synthesis was measured at the end of an experiment, the medium was removed and a leucine-free HEPES medium containing 1 μCi/mL [^3^H]Leu was added. After a 15 min incubation at 37 °C, the medium was removed and 5% (*w*/*v*) trichloroacetic acid (TCA; 100807 Merck, Saint Louis, MO, USA) was added. The cells were after 5 min washed once in TCA and dissolved in 0.1 M KOH. The protein synthesis was then quantified by measuring the amount of [^3^H]Leu incorporated into proteins by β-counting on a Tri-Carb 2100TR Liquid Scintillation Analyzer (Packard BioScience, Meriden, CT, USA).

### 4.3. Experiments with Radioactive Sulfate

Sulfation of ricin-sulf1 (RS1) (4 μg/mL) and ricin-sulf2 (RS2) (15 µg/mL) was in principle carried out as previously described [28]. Specific incubation times with ^35^ SO_4_^2−^ (0.2 mCi/mL) and inhibitors are specified in the figure legends. In all cases except for the experiment where release of A-chain was measured, the labeled ricin was analyzed by running reducing gels. At the end of the incubation with RS1 and RS2, surface-bound toxin was removed by treating the cells with 0.1 M lactose in HEPES-buffered medium (MEM without bicarbonate but supplemented with 20 mM HEPES, 2 mM L-alanyl-L-glutamine, 100 U/mL penicillin, and 100 μg/mL streptomycin) for 5 min at 37 °C. The cells were washed a second time in the same solution, then in ice-cold PBS (1.1 mM NaH_2_PO_4,_ 5.5 mM Na_2_HPO_4_, 138.6 mM NaCl; pH 7.4) and lysed in lysis buffer (0.1 M NaCl, 10 mM Na_2_HPO_4_, 1 mM EDTA. 1% TritonX-100 supplemented with cOmplete^TM^ Protease Inhibitor Cocktail (05056489001, Roche Diagnostics, Mannheim, Germany), plus 60 mM n-octyl-β-glucopyranoside; pH 7.4). The lysates were cleared by centrifugation and immunoprecipitation with anti-ricin or anti-ricin-A-chain on Protein A Sepharose^®^ beads (17-0963-03 GE Healthcare) was performed overnight at 4 °C. Then, the immunopreciptates were washed twice in 0.35% Triton X-100 in PBS, resuspended in Laemmli sample buffer (161-0747, Bio-Rad Laboratories Inc, Hercules, CA, USA) with or without 100 mM dithiothreitol (DTT), and boiled for 5 min. Proteins in these samples were separated by SDS-PAGE, blotted onto a PVDF membrane, and visualized by digital autoradiograpy by using a phosphor imaging screen (Imaging Screen-K (Kodak), Bio-Rad Laboratories Inc.) and the Molecular Imaging PharosFX system (Bio-Rad Laboratories Inc., Hercules, CA, USA). The intensity of the bands was quantified using the Quantity One 1-D Analysis Software (Biorad Laboratories Inc., Hercules, CA, USA). For determination of the total amount of protein sulfation, proteins from the supernatant left after immunoprecipitation were precipitated with 5% TCA. The TCA precipitate was dissolved in 0.1 M KOH and the radioactivity was measured by liquid scintillation counting using a Tri-Carb 2100TR Liquid Scintillation Analyzer (Packard).

### 4.4. Ricin Endocytosis, Recycling and Degradation

These parameters were measured as described by Lingelem et al. [28], with incubation times as specified in the figure legends. For these experiments, ricin was ^125^I-labeled by using Pierce Iodination Tubes (cat. No 28601, Thermo Fisher Scientific, Rockford, IL, USA) according to the protocol from the manufacturer. Ricin endocytosis was measured as the amount of lactose-resistant ^125^I-ricin in percent of total cell-associated ricin as previously described [28]. The radioactivity was measured using a Hidex Automatic Gamma Counter (Hidex). In principle, degradation of ricin was measured by first incubating with ^125^I-ricin for 20 min, removing the surface-bound toxin with 0.1 M lactose in HEPES-buffered medium, and then chasing the toxin for 2 h in the presence of 1 mM lactose to inhibit rebinding and further uptake of the toxin. At the end of this incubation, the total and the TCA-precipitable radioactivity was measured in the medium, the cells were dissolved in 0.1 M KOH, and the radioactivity in the cells was measured. The pellet from the medium contained the recycled ricin, whereas the non-precipitable radioactivity represents ricin that was degraded during this time-period of incubation. For further details, see [28].

### 4.5. Transferrin Binding and Endocytosis

Transferrin was ^125^I-labeled by using Pierce Iodination Tubes (cat. No 28601, Thermo Fisher Scientific) according to the protocol from the manufacturer. The experiments were carried out on cells growing in 24-well disposable trays. The ^125^I-transferrin used (200 ng/mL; ~25,000 cpm/ng) was added after incubation with inhibitors as described in the figure legend.

### 4.6. Statistical Data Analysis

The differences between means for two groups were determined using two-tailed Student’s *t*-test. The level of significance was set as follows: * *p* ≤ 0.05, ** *p* ≤ 0.005 compared to control unless otherwise stated. Unless otherwise specified, error bars represent SD.

## Data Availability

Data are contained within the article or Supplementary Materials.

## Supplementary Materials

The following supporting information can be downloaded at: https://www.mdpi.com/article/10.3390/toxins14050360/s1. Figure S1: EACC reduced cell sensitivity to ricin in PC3 and HeLa cells; Figure S2: MG132 did not affect ricin toxicity and EACC-induced protection; Figure S3: 8-Br-cAMP and crizotinib did not change the EACC-induced protection against ricin; Figure S4: MRT increased HEp-2 and HeLa cell sensitivity to ricin; Figure S5: MRT68601-induced changes in ricin binding, uptake, and transport to Golgi; Figure S6: Effect of EACC and MRT68601 on binding and uptake of transferrin.
