# Supplementary material for: Modulation of Ricin Intoxication by the Autophagy Inhibitor EACC
Source: Toxins (Basel). 2022 May 22;14(5):360. doi: 10.3390/toxins14050360 (PMC9145485; doi:10.3390/toxins14050360)
Supplement: Supplementary file 1 [file toxins-14-00360-s001.zip › toxins-1724147-supplementary.pdf]

# Supplementary Materials: Modulation of Ricin Intoxication by the Autophagy Inhibitor EACC

**Kirsten Sandvig, Simona Kavaliauskiene, Anne Grethe Myrann, Tore Geir Iversen and Tore Skotland**

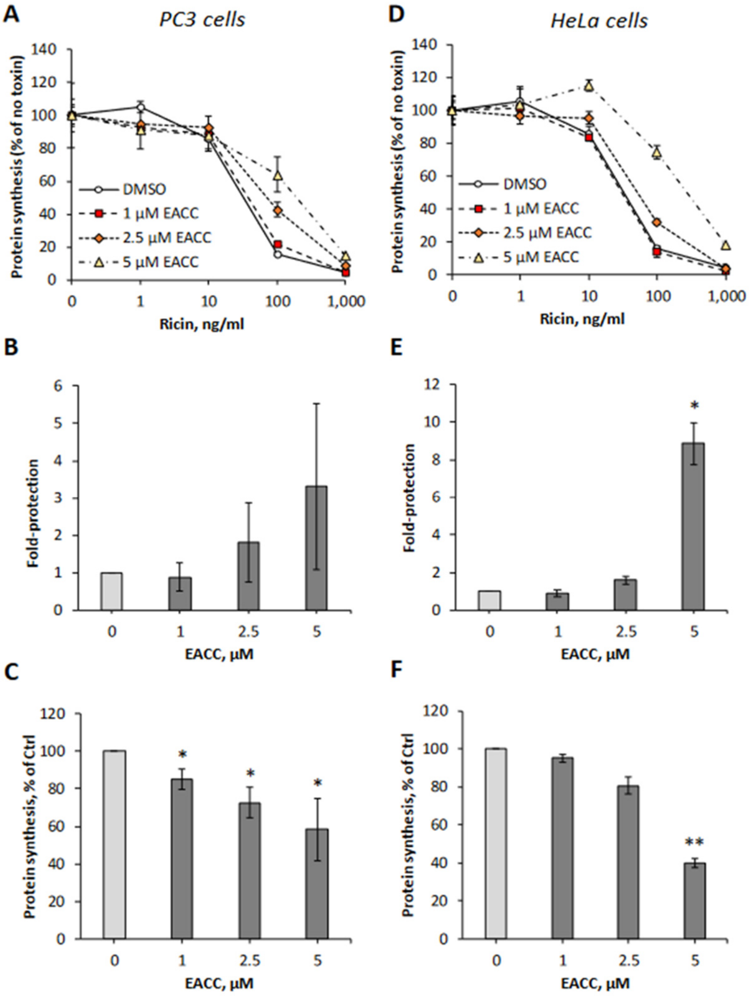


**Figure S1.** EACC reduces cell sensitivity to ricin in PC3 and HeLa cells. (A-C) PC3 cells were treated with EACC for 30 min and subsequently incubated with increasing concentrations of ricin for 3 h, before protein synthesis was measured. A representative experiment is shown in (A). (B) Fold protection was calculated as an increase in IC50 in EACC treated samples compared to control (DMSO treated cells). (C) Protein synthesis in the cells treated with 3.5 h (30 min + 3 h when toxin is added to other wells) with EACC. (D-F) HeLa cells were treated with EACC for 2 h and subsequently incubated with increasing concentrations of ricin for 3 h, before protein synthesis was measured. A representative experiment is shown in (D). (E) Fold protection was calculated as in an increase in IC50 in EACC treated samples compared to control (DMSO treated cells). (F) Protein synthesis in the cells treated with 5 h (2 h + 3 h when toxin is added to other wells) with EACC. N ≥ 3 for PC3 cells and N ≥ 2 for HeLa cells.

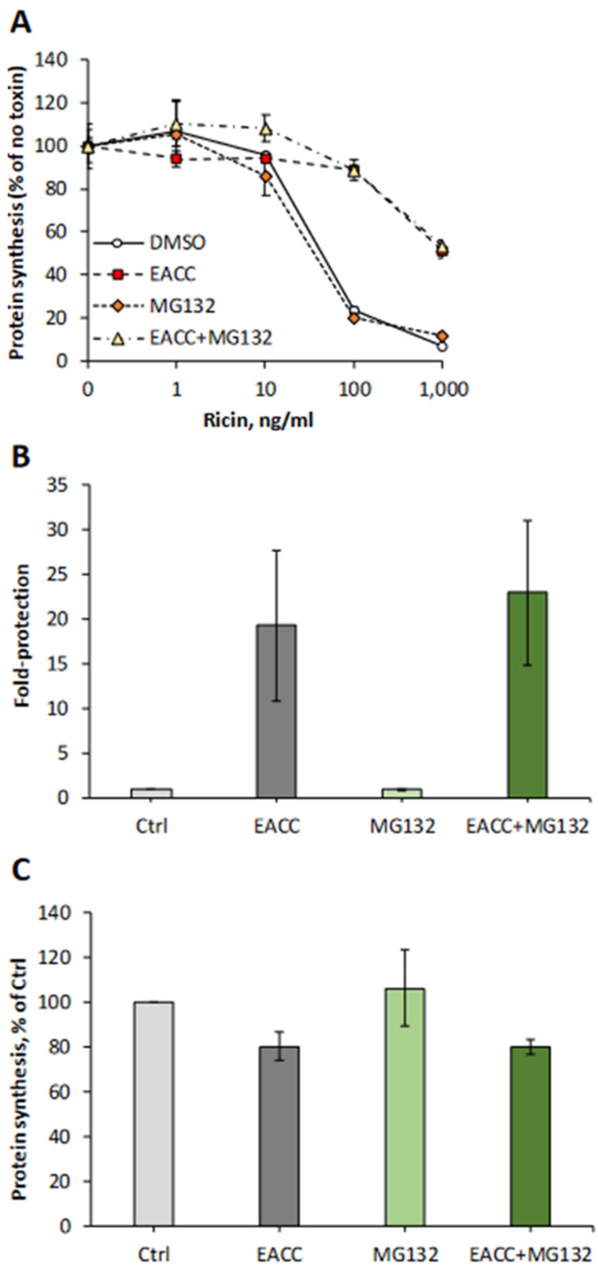


**Figure S2.** MG132 does not affect ricin toxicity and EACC-induced protection. HEp-2 cells were treated with EACC for 2 h and subsequently incubated with increasing concentrations of ricin and 10 μM MG132 in half of the wells for 3 h, before protein synthesis was measured. A representative experiment is shown in (A). (B) Fold protection was calculated as an increase in IC50 in inhibitor-treated samples compared to control (DMSO treated cells). (C) Protein synthesis in the cells treated with inhibitors for 5 h (2 h + 3 h when toxin is added to other wells). N=2.

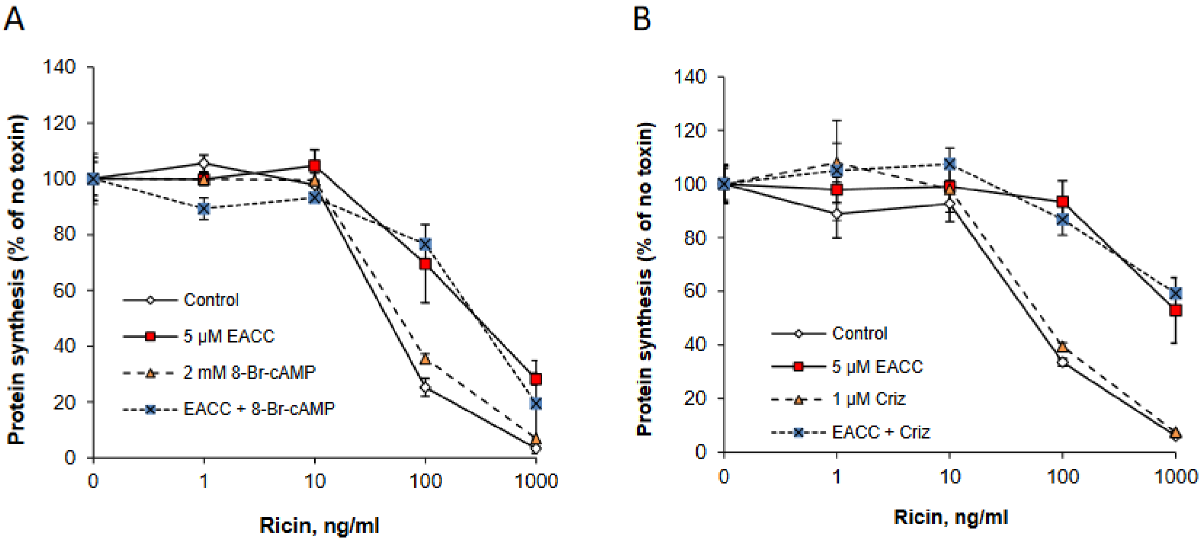


**Figure S3.** 8-Br-cAMP and crizotinib do not change the EACC-induced protection against ricin**.** (A) HEp-2 cells were treated with or without 5μM EACC, 2 mM 8-Br-cAMP or the combination for 2 h before increasing concentrations of ricin were added. Protein synthesis was measured 3 h later as described in Materials and Methods. (B) HEp-2 cells were incubated with and without 1μM crizolinib for 30 min before addition of 5μM EACC to some of the cells. 2 h later increasing concentrations of ricin were added and the protein synthesis was measured after 3 h in the presence of the toxin. The error bars show deviations between duplicates in a representative experiment. N=2.

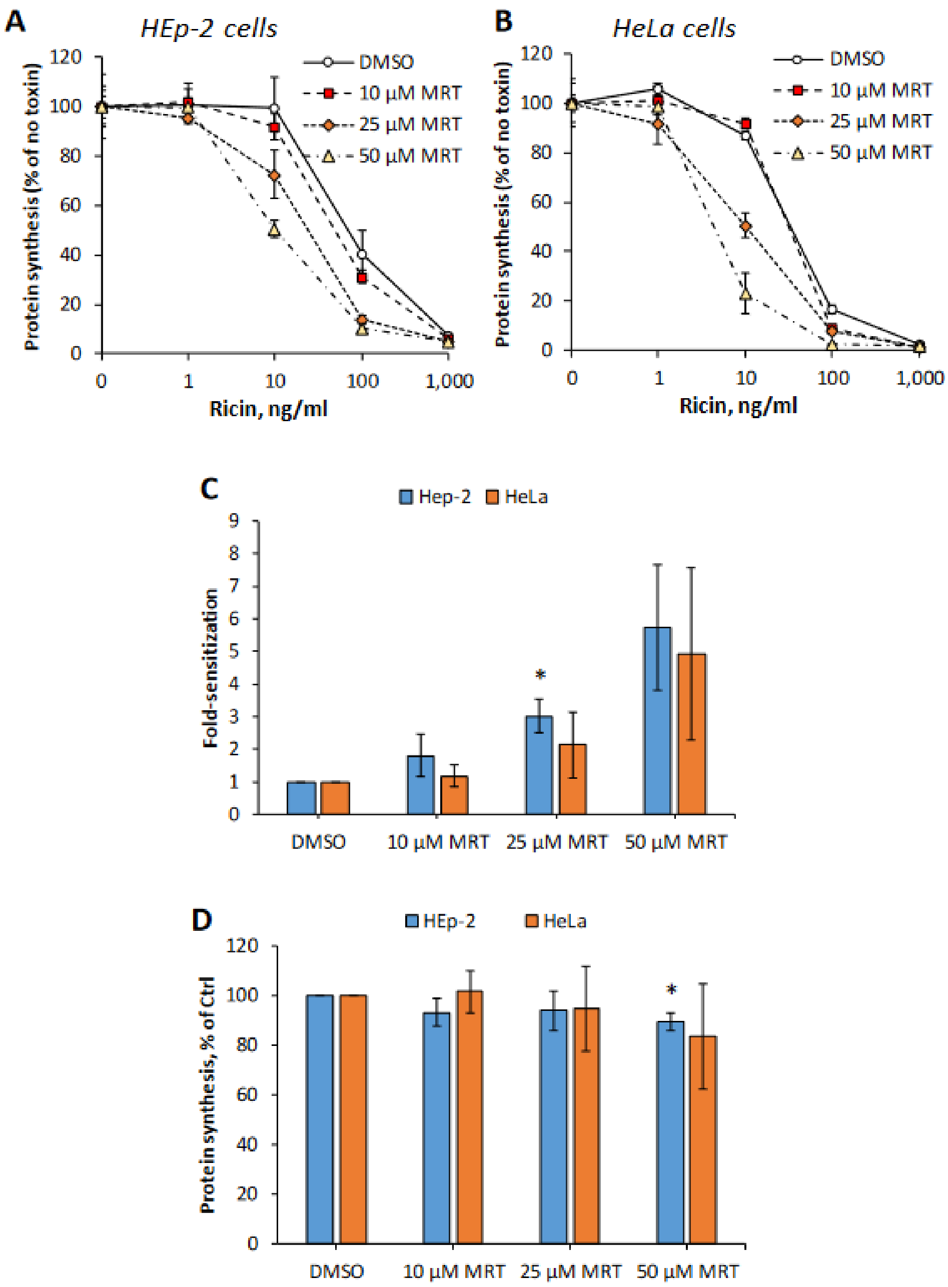


**Figure S4.** MRT increases HEp-2 and HeLa cell sensitivity to ricin. HEp-2 and HeLa cells were treated with MRT for 1 h and subsequently incubated with increasing concentrations of ricin before protein synthesis was measured. A representative experiment is shown in (A) for HEp-2 cells and in (B) for HeLa cells. (C) Fold sensitization was calculated as a reduction in IC50 in inhibitor-treated samples compared to control (DMSO-treated cells). (D) Protein synthesis in the cells treated with inhibitors for 4 h (1 h + 3 h when toxin is added to other wells). N =3.

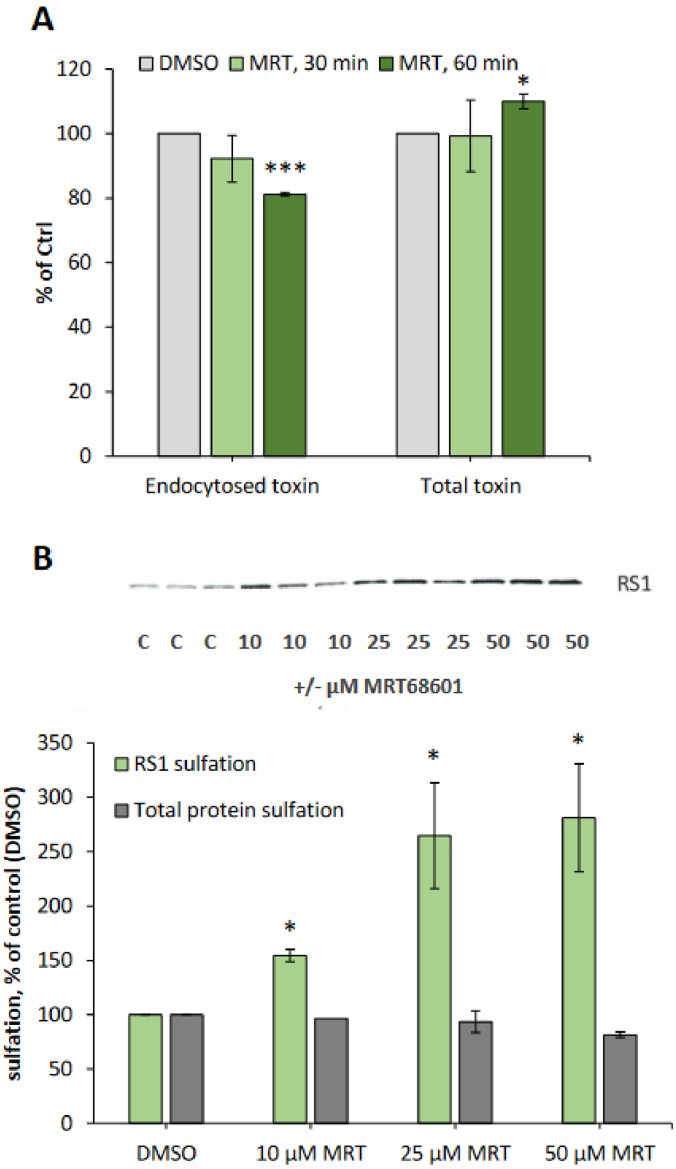


**Figure S5.** MRT68601-induced changes in ricin binding, uptake and transport to Golgi. (A) HEp-2 cells were treated with 0.1% DMSO or 25µM MRT68601 for 30 min or 60 min followed by incubation with $^{125}$I-ricin for 20 min. Ricin endocytosis was measured as described in the Materials and Methods. (B) HeLa cells were treated with 0.1% DMSO or MRT68601 (10, 25 or 50 µM) for 1 h and then they were subjected to sulfation assay with ricin-sulf1 (RS1). Radioactive sulfate was added 2 h before MRT68601 and the cells were incubated with RS1 for 3 h before determination of radioactive labeling of RS1. The upper figure shows an autoradiogram of one representative experiment, and the lower figure shows the quantification of RS1 sulfation expressed as percentage of control (DMSO), N≥2.

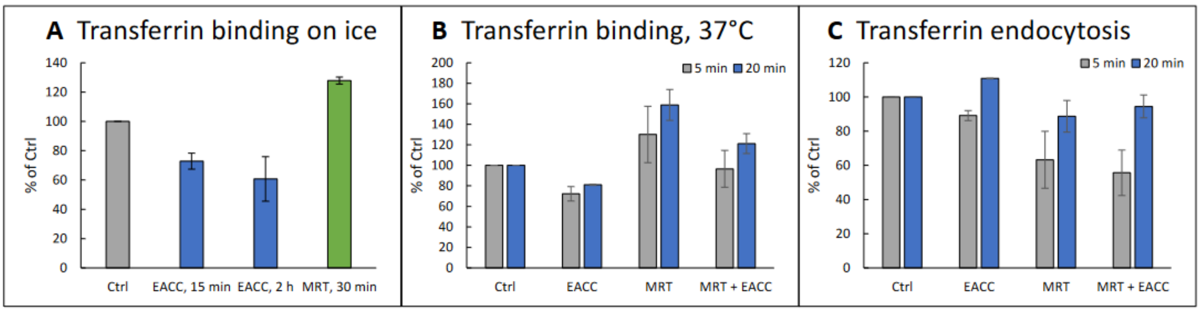


**Figure S6.** Effect of EACC and MRT68601 on binding and uptake of transferrin. (A) HEp-2 cells were washed once with HEPES-buffered medium and then treated with 5 µM EACC for 15 min or 2 h, or with 25 µM MRT68601 for 30 min at 37 °C. Following inhibitor treatment, cells were cooled on ice for 10 min and then $^{125}$I-transferrin was added, and the cells were kept on ice for additional 30 min. The cells were then washed three times with cold PBS, lysed in 0.1 KOH and gamma radiation was measured. (B, C) Hep-2 cells were treated with 25 µM MRT68601 for 30 min, then 5 µM EACC was added and incubation was continued for 2 h at 37 °C. $^{125}$I-transferrin was added directly to medium with inhibitors and cells were incubated at 37 °C for 5 or 20 min, then washed three times with cold PBS and incubated with 2mg/ml pronase in HEPES-buffered medium for 1 h on ice. The medium was collected and centrifuged, and the radioactivity was counted in the pellet and the supernatant. Total counts (pellet + supernatant) represent total cell-associated $^{125}$I-transferrin (B). The endocytosed transferrin (pellet) was calculated as a percentage of total cell-associated transferrin and normalized to control (C). Each independent experiment (N=2) was performed with duplicates.
